# Comprehensive multi-omics reveals dynamic chromatin changes and gene regulatory networks during duck folliculogenesis

**DOI:** 10.1186/s40104-026-01393-z

**Published:** 2026-04-30

**Authors:** Zhen Li, Yunxiao Sun, Dandan Sun, Ning Yang, Zhongtao Yin, Zhuocheng Hou

**Affiliations:** Frontiers Science Center for Molecular Design Breeding (MOE), State Key Laboratory of Animal Biotech Breeding, National Engineering Laboratory for Animal Breeding, Key Laboratory of Animal Genetics, Breeding and Reproduction of the Ministry of Agriculture, College of Animal Science and Technology, China Agricultural University, Beijing, China

**Keywords:** Bird, *Cis*-regulatory map, Folliculogenesis, Regulatory network, Three-dimensional

## Abstract

**Background:**

Follicular development is a prerequisite for vertebrate reproduction, and it is precisely regulated by complex genomic conformations and regulatory elements. However, the dynamic changes in the interaction between the three-dimensional genome and regulatory elements of granulosa cells (GCs) during avian follicular development are still unclear. Here, we integrated RNA sequencing, ATAC sequencing, CUT&Tag, and Hi-C of GCs in 7 stages of Pekin ducks (*Anas platyrhynchos* *domestica*) to construct a high-resolution three-dimensional *cis*-regulatory map of follicular development, revealing the chromatin dynamics basis of avian folliculogenesis.

**Results:**

Our integrative analysis reveals that H3K27ac dynamics, rather than chromatin accessibility alone, are strongly associated with the stage-specific transcriptional increase of follicle selection and maturation. We identified enhancers and super-enhancers (SEs) that are significantly correlated with the expression of key follicular genes. Regarding 3D genome organization, we observed that topologically associating domains (TADs) remained largely stable, serving as a structural scaffold. However, stage-specific boundary changes coincided with the transcriptional alterations of key regulator genes. Furthermore, we inferred putative gene regulatory networks (GRNs) comprising 46 core transcription factors (TFs) predicted to be closely linked to follicular development. Finally, comparative analysis highlighted both the conservation and species-specificity of these regulatory elements between birds and mammals.

**Conclusions:**

Our study provides an integrative, multi-omics resource that offers novel insights into the epigenomic landscape of duck follicular development. The resulting dataset and regulatory map establish a valuable foundation for further mechanistic studies of folliculogenesis and for understanding regulatory divergence across species.

**Graphical Abstract:**

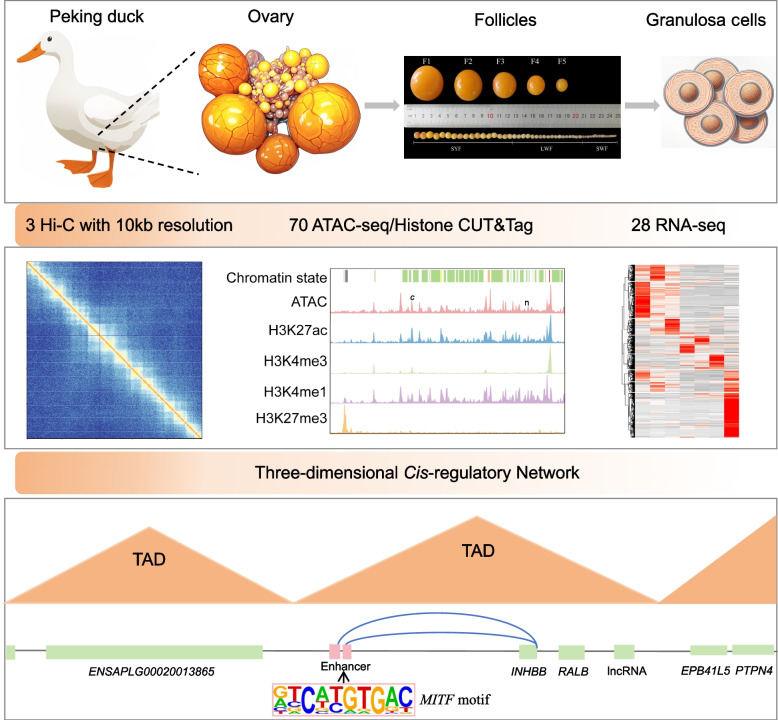

**Supplementary Information:**

The online version contains supplementary material available at 10.1186/s40104-026-01393-z.

## Background

Reproduction is a basic life activity, and the ovary is an important reproductive organ of vertebrates. In vertebrates, the ovary contains follicles at different developmental stages [1, 2]. Extensive studies have shown that epigenetic regulation plays a pivotal role in controlling follicular development in mammals [3, 4]. Viviparous animals (represented by humans) and oviparous animals (represented by birds) exhibit significant differences in ovarian morphology, follicular structure, follicular development stages, and sex hormone secretion [2, 5–10]. It is well known that birds have excellent reproductive performance, especially domesticated poultry. Their eggs supply a substantial, low‑cost source of high‑quality protein worldwide [11]. Thus, birds represent not only a successfully domesticated agricultural species but also a valuable model for investigating vertebrate follicular development and evolution [12]. This shows the indispensable role of animal models in bridging basic biological discovery and translational applications [13].

Follicles are located within the ovarian cortex and are composed of oocytes, GCs, theca cells, etc. [14]. Follicles are classified by diameter into three main categories: prehierarchical follicles, including small white follicles (SWFs), large white follicles (LWFs), and small yellow follicles (SYFs); hierarchical follicles (from F5/F6 to F1); and postovulatory follicles (POFs) [15]. Multiple follicles coexist at each prehierarchical stage, whereas hierarchical follicles follow a strict hierarchical development [16]. Follicle selection and ovulation (the rupture of a mature follicle in the ovary) constitute critical stages governing egg‑production output. Only one selected follicle enters the hierarchical stage from prehierarchical follicles each day, and these follicles are ultimately destined for ovulation. As an essential component of the follicle, GCs play crucial roles in follicular growth, development, and ovulation via their proliferation, differentiation, and bidirectional communication with the oocyte [14, 17–19].

In birds, follicular development directly determines laying rate. Despite an ovarian reserve of hundreds of thousands of primordial follicles in the avian embryo, fewer than 1% ultimately mature and ovulate [16]. Deciphering the molecular regulatory mechanisms underlying follicular development to improve ovulation efficiency represents a pivotal challenge. Previous work in chickens revealed that gene expression in the SWF, F1, and POF stages correlates with H3K27ac activity, suggesting that enhancers may drive changes in gene expression during follicular development [20]. Additional studies have shown that chromatin accessibility and RNA N^6^‑methyladenosine (m^6^A) modification also influence follicular development and egg production [21–23]. However, the dynamic changes in epigenetic modifications and gene expression during follicle selection and maturation (the F5/F6 stage develops into the F1 stage) remain unclear, and it is unknown whether birds employ conserved regulatory mechanisms for follicular development.

In this study, we constructed a comprehensive three‑dimensional *cis*‑regulatory landscape of duck follicular development by integrating multiple transcriptomic and epigenomic datasets. Overall, our study provides systematic evidence for the regulatory role of epigenomics in avian follicular development and offers potential directions for future research on epigenetic regulation of follicle development. Emerging biotechnologies, including mRNA vaccines and nanovaccines, may offer novel tools for the targeted modulation of reproductive processes [24, 25].

## Methods

### Animals and sample collection

To minimize variability relative to ovulation, all 30 ducks were selected from a high-performing flock (peak laying period, > 90% production rate) to ensure regular daily ovulation. GCs were isolated from follicles at various stages, including SWFs, LWFs, SYFs, F5, F3, F1, and POFs. On average, we observed 50.5 ± 19.1 pre-hierarchical follicles (> 1 mm) per duck. We provide typical diameters for F1 to F5 follicles observed from a representative individual (F1: ~ 3.8 cm; F2: ~ 3.3 cm; F3: ~ 2.8 cm; F4: ~ 2.2 cm; F5: ~ 1.4 cm). The detailed procedure is as follows: secure the follicle with forceps so that the transparent, non‑porous stigma faces upward, and make a quick incision along the stigma line. Release the yolk from inside the follicle. Once most of the yolk has been expelled, rapidly turn the follicle inside out. Then, peel away the transparent GC layer situated between theca cells and the yolk [20, 26]. All isolated GC samples were immediately frozen in liquid nitrogen and stored at −80 °C for subsequent analysis, including RNA-seq, Hi-C, ATAC-seq, and CUT&Tag.

### Library construction and sequencing

RNA-seq, ATAC-seq, CUT&Tag (H3K4me3, H3K4me1, H3K27ac, and H3K27me3), and Hi-C experiments were conducted on the frozen tissue samples. There were two biological replicates for both the ATAC-seq and histone CUT&Tag in each stage, and RNA-seq had four replicates.

For RNA-seq, libraries were constructed following the VAHTS Universal V6 RNA-seq Library Prep Kit for Illumina® (NR604-01/02) protocol. Different indexing tags were selected for library construction. Upon completion, libraries were quantified, and the insert size was assessed using Qubit and Agilent 2100 systems. The effective library concentration was precisely determined by quantitative PCR (qPCR) with the Bio-Rad iQ SYBR Green Kit. Sequencing was then carried out on the HiSeq X Ten platform.

For ATAC-seq, nuclei were extracted from 100,000 cells per reaction and incubated with Tn5 transposase and tagging buffer at 37 °C for 30 min (Vazyme Biotech, TD711-01). DNA was then purified using a PCR purification kit. The amplification library was generated through PCR cycles with the following conditions: 72 °C for 3 min, 95 °C for 3 min, followed by 10–15 cycles of 98 °C for 10 s and 60 °C for 5 s, and finally 72 °C for 1 min. After the PCR reaction, the library was purified using DNA Clean Beads (Vazyme #N411). Sequencing was conducted on the Illumina Novaseq platform.

For CUT&Tag, approximately 50,000 nuclei per reaction were gently resuspended in 50 μL of antibody buffer (Vazyme Biotech, TD904-01) containing the respective primary antibodies and incubated overnight at 4 °C. After centrifugation to remove the primary antibody solution, nuclei were incubated with a secondary antibody in wash buffer for 1 h. Following two washes, nuclei were incubated with pre-assembled pA/G-Tnp complexes for 1 h. After two additional washes, tagmentation was performed by incubating nuclei in labeling buffer at 37 °C for 1 h. The reaction was stopped, and the DNA libraries were amplified using the same PCR program as for ATAC-seq. The final libraries were purified with DNA Clean Beads (Vazyme #N411) and sequenced on an Illumina NovaSeq platform.

For Hi-C, GCs at different developmental stages were homogenized in liquid nitrogen and cross-linked at room temperature with 4% formaldehyde for 30 min. Following cross-linking and lysis, the DNA was digested with the restriction enzyme MboI at 37 °C. The DNA ends were biotinylated and incubated at 37 °C for 45 min, followed by heat inactivation of the enzyme. T4 DNA ligase was added to facilitate DNA ligation, and incubation occurred for 1–2 h. After ligation, proteinase K was added for reverse cross-linking, and incubation was performed at 65 °C for 1–2 h. The DNA fragments were then purified and dissolved in water. Unligated ends were removed, and the purified DNA was fragmented to 350–500 bp. The DNA ends were repaired before isolating biotinylated DNA fragments on dynabead® M-280 Streptavidin (Life Technologies, 60210). Each library was sequenced on the Illumina HiSeq X Ten platform with 150 bp paired-end reads.

### RNA-seq data analysis

The raw data were initially processed using fastp (0.20.1) [27] to remove adapter sequences and low-quality reads, obtaining high-quality clean data. Clean reads were then aligned to the *Anas platyrhynchos* genome (GCA_008746955) using HISAT2 (4.8.2) [28, 29]. Gene expression was quantified using HTSeq, and differential gene expression analysis across developmental stages was performed with DESeq2 (1.44.0) [30, 31]. Genes with adjusted FDR < 0.05 and absolute log_2_ fold change > 1 were defined as differentially expressed genes (DEGs). The DEGs were further analyzed using the ClusterProfiler package (4.12.6), and a comparative analysis was conducted with the Kyoto Encyclopedia of Genes and Genomes (KEGG) database (https://www.genome.jp/kegg/) [32]. Gene Ontology (GO) functional annotation was performed using the default settings of Metascape (http://metascape.org) [33].

### CUT&Tag and ATAC-seq data analysis

Raw reads were processed using fastp (v0.20.1) [27]. Clean reads were aligned to the duck genome (GCA_008746955-1) using Bowtie2 [28, 34]. SAMtools (v1.15.1) [35] was used to sort and filter aligned reads, while Picard [36] was utilized to remove duplicate reads. DeepTools (v3.5.0) provides bamCoverage converted to bigwig files [37].

Peak calling was performed with MACS2 (v2.2.7.1) [38]. For ATAC-seq data, the peak calling parameters were set to “--nomodel --shift 100 --extsize 200 -B”. For the histone marks, narrow peaks (H3K27ac, H3K4me3) were called using “-q 0.1 -B -f BAMPE”, while broad peaks (H3K27me3, H3K4me1) were called with “--broad --broad-cutoff 0.1 -f BAMPE -q 0.1”. For the quantification of ATAC-seq and CUT&Tag data, DiffBind (v3.14.0) [39] was employed to generate read counts under reference peaks, normalized to counts per million. This approach facilitated the identification of different peaks. Heatmaps centered on transcription start sites (TSS) were generated using ComputeMatrix and PlotHeatmap [37].

### Hi-C data analysis

Hi-C reads were mapped to the duck genome using HiC-Pro (v3.1.0) [40]. The proportion of bins with more than 1,000 interactions at a 10 kb resolution in the three stages of Hi-C was calculated. And it was over 80%, indicating that the resolution of Hi-C data was ~ 10 kb [41]. Principal component analysis (PCA) was conducted using the runHiCpca.pl script from HOMER [42] to further delineate active (“A” compartment) and inactive (“B” compartment) chromatin regions across the genome. TAD structures were defined at 20 kb resolution using the hicPlotTADs function from HiCExplorer (v3.7.2) [43]. Specific TAD boundaries were identified using a previously reported method [44]. Briefly, to compare boundaries between two stages, we extracted signals from 10 bins upstream and downstream (± 200 kb) centered on each boundary. The Spearman correlation coefficient was calculated between boundary pairs from adjacent stages. For robust comparison, this calculation was repeated 1,000 times using randomly selected bins to generate a random distribution of correlation coefficients. A boundary was defined as a stage-specific boundary if it met two criteria: 1) it was identified only in one stage, and 2) its correlation with any boundary from adjacent stages was not significant.

### Chromatin state annotation and state variability

Chromatin states were predicted by integrating ATAC-seq and CUT&Tag signals from two biological replicates across all seven stages using ChromHMM (v1.25) [45]. A 15-state model was learned, and states were annotated based on the enrichment of epigenomic marks surrounding TSS [46–48]. To investigate chromatin state changes across different tissues, BEDtools (v2.29.2) [49] was used to identify differential regulatory elements between adjacent stages.

### Construct GRNs

Utilizing Hi-C data from SYF, F5, and F1 stages, TAD structures were delineated. The GRN was constructed by integrating 3D chromatin interactions with motif analysis. First, putative enhancer-gene interactions were inferred by calculating the Pearson correlation coefficient within each TAD (FDR < 0.05). Second, TF binding motifs were enriched within these enhancer regions using HOMER (*P* < 1e-5), establishing TF-Enhancer pairs. Finally, we integrated these datasets to build a TF-Enhancer-Target GRN, illustrating how TFs regulate stage-specific gene expression. Furthermore, the structural robustness of the inferred networks was evaluated by random edge removal, yielding high stability scores (> 0.9). Permutation tests (*n* = 1,000) further confirmed that the inferred topologies were significantly distinct from random networks (*P* < 0.05). Additionally, a core gene network was further extracted by focusing on: (1) These TFs have binding motifs significantly enriched in dynamic enhancers (enhancers with changing activity between stages); (2) The TFs are DEGs and are co-regulated with their target genes in the expression clusters.

### SEs and SSs prediction

SEs and super-silencers (SSs) were identified using ROSE [50] based on the H3K27ac and H3K27me3 signals from each sample. SEs identified in replicates were merged. Stage-specific SEs and SSs with differential activity between adjacent stages were determined using Bedtools (v2.29.2) [49]. SE activity was clustered using k-means analysis in the ComplexHeatmap package (2.20.0) [51]. GO and KEGG enrichment analyses were performed on genes associated with SEs using Metascape [33].

### Distal enhancer-gene interactions

Chromatin loops were identified using the hicDetectLoops tool in HiCExplorer software (v3.7.2). In this study, loop structures were identified at various resolutions (10, 20, 25 kb), and then the hicMergeLoops tool was used to integrate these loops identified at all resolutions into a 25 kb range. A total of 3,192, 3,206, and 4,093 loops were identified in the SYF, F5, and F1 stages, respectively. The loop structures were then used to capture distal regulatory relationships. Enhancers and promoters overlapping with these anchors were identified using Bedtools (v2.29.2).

### Conservation analysis

ChIP-seq data for pre-ovulatory H3K27ac from chickens [20], humans (PRJNA1052695), pigs (PRJNA1113520), and mice [52] were downloaded from NCBI. Bam and peaks files were generated as described above, and enhancers and SEs were identified using ROSE [50]. The LiftOver tool [53] was used to map enhancer coordinates from chicken, mouse, and pig to the human genome (hg38), and from chicken to the duck genome. Enhancers that could be mapped to human or duck genomic positions were considered to be conserved at the sequence level. Furthermore, enhancers were considered functionally conserved if the corresponding homologous sequences overlapped with enhancers. Gene orthologs between five species were retrieved from Ensembl BioMart. Motif enrichment analysis of the enhancers was performed using HOMER, resulting in the identification of overlapping TFs.

## Results

### Transcriptomic and epigenomic profiling of GCs during follicular development in ducks

**Fig. 1:**
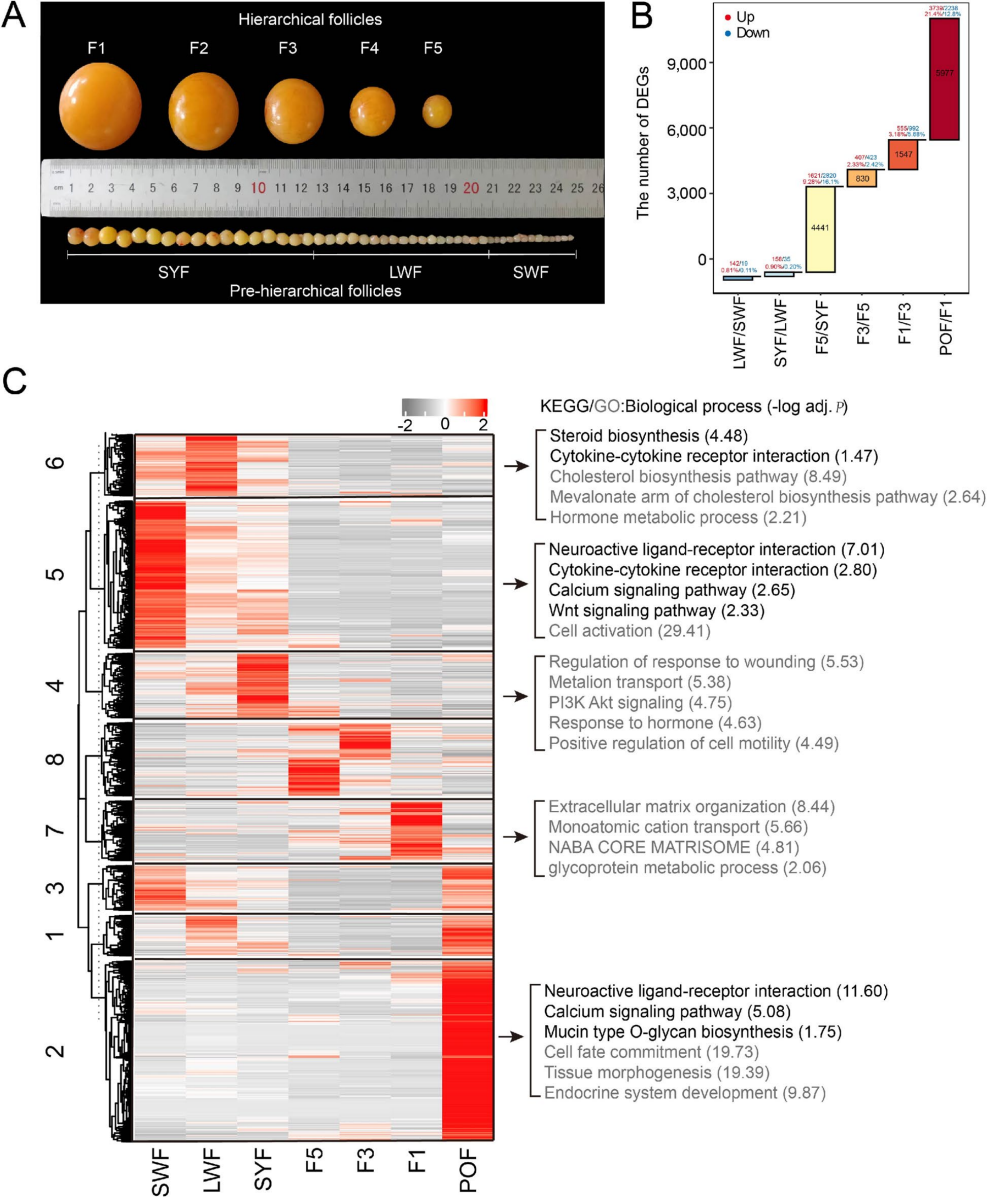
Transcription and epigenome profiling of GCs during follicular formation. **A** The diagram of ovarian follicle development in the duck. **B** Number and percentage of DEGs between consecutive stages. DEGs were defined as |log_2_FC| ≥ 1 and FDR < 0.05. **C** K-means Cluster analysis alongside KEGG and GO enrichment analysis for each cluster gene

To explore the transcriptional and chromatin dynamics during follicular development, we generated 28 RNA sequencing libraries, 14 Assay for ATAC-seq, 56 CUT&Tag for histone modification analysis (H3K27me3, H3K4me3, H3K4me1, and H3K27ac), and 3 Hi-C experiments in duck follicular development. These stages included SWFs, LWFs, SYFs, preovulatory follicles (F5, F3, and F1), and POFs (Fig. 1A). We obtained a total of 3.5 billion mapped reads and detected an average of 31,030, 24,269, 23,815, 60,772, and 42,109 peaks for ATAC, H3K4me3, H3K27ac, H3K4me1, and H3K27me3 (Fig. S1A, B and Tables S1, S2). The PCA and correlation results confirmed high reproducibility among biological replicates within each stage (Fig. S1C–E).

To characterize gene expression dynamics during follicular development, we identified 8,137 DEGs. The proportion of DEGs was significantly higher between SYF and F5 (~ 25.42% of genome-wide genes) and between F1 and POF (~ 34.21% of genome-wide genes) compared to other stage transitions (*P* < 0.01, Fisher's exact test) (Fig. 1B, Tables S3 and S4). As expected, this indicated that significant changes have occurred in the follicle selection and ovulation. We grouped the DEGs into eight distinct clusters using K-means clustering analysis. GO and KEGG enrichment analyses were performed to gain insights into the biological processes associated with each cluster (Fig. 1C). Clusters 4, 5, and 6 showed high expression levels in the hierarchical stages and were primarily involved in hormone synthesis and signal transduction, such as Steroid biosynthesis, Cytokine-cytokine receptor interaction, and Wnt signaling pathway. These results highlight the distinct biological functions of GCs across folliculogenesis.

### Specific chromatin state changes during follicular development

**Fig. 2:**
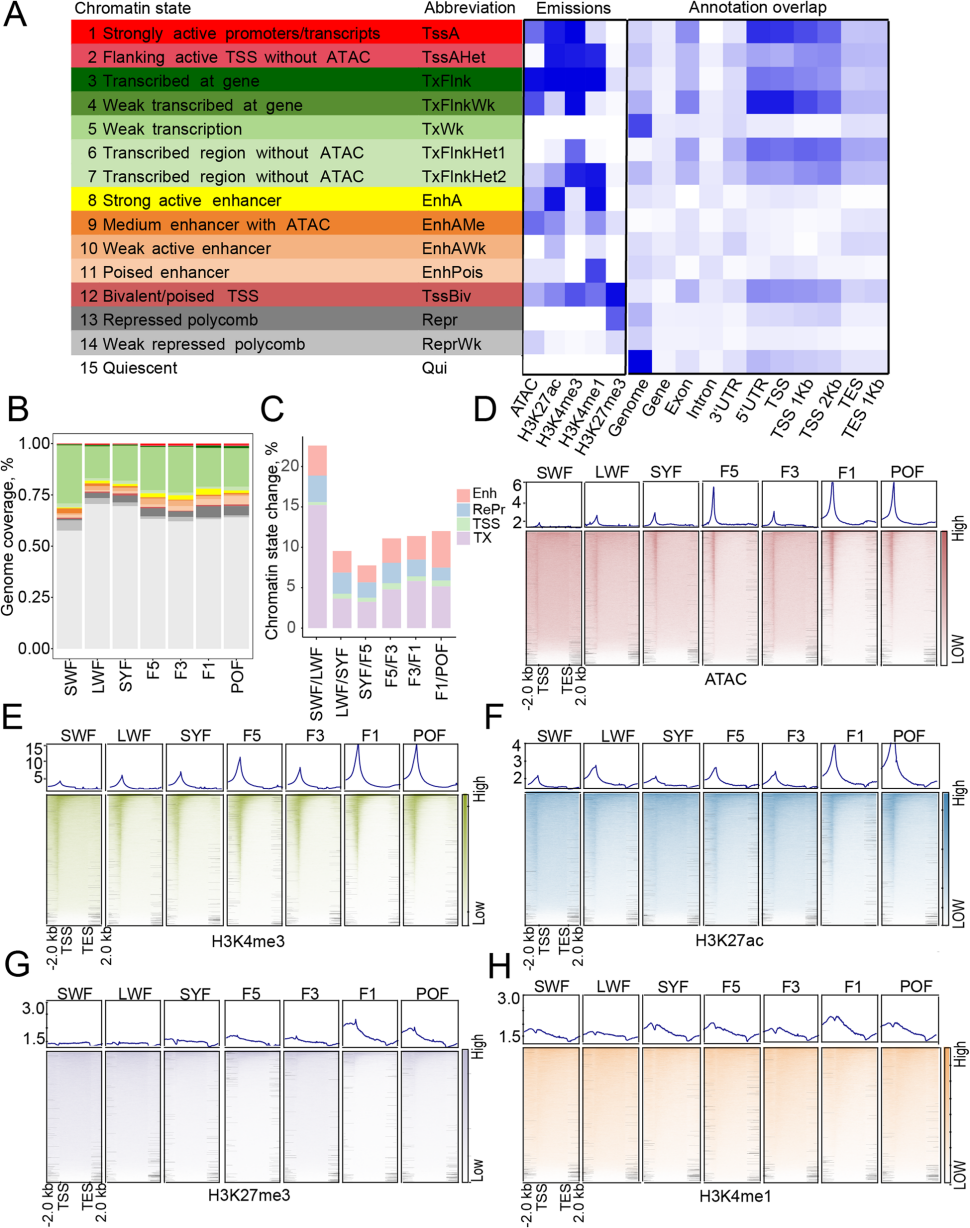
Dynamics of histone modifications during follicular development. **A** Definition of 15 predicted chromatin states (left), the emission probability of five epigenetic marks for each chromatin state (middle), and the average enrichment of genome-annotated chromatin states, including genomic regions such as genes, exons, introns, and TSS/TES 1 kb (± 1 kb around TSS and TES) across different stages (right). **B** Coverage of chromatin states at each developmental stage. Color coding corresponds to the states defined in panel A.** C** Relative change in state coverage between consecutive stages, normalized to total genome size. **D–H** Dynamics of chromatin accessibility and histone modifications throughout follicular development

To elucidate the epigenetic basis of gene expression changes, we analyzed chromatin state dynamics using ChromHMM, integrating four histone modifications and chromatin accessibility across 7 stages. This defined 15 chromatin states, encompassing 2,176,920 regulatory elements (excluding Qui) (Fig. 2A and Table S5). These elements were classified as follows: promoters (TssA, TssAHet, and TssBiv, covering 1.50% of the genome), enhancers (EnhA, EnhAMe, EnhAWk, EnhPois, comprising 6.26% of the genome), TSS-proximal transcription regions (TxFlnk, TxFlnkWk, TxWk, TxFlnkHet, constituting 22.47% of the genome), silencers (Repr and ReprWk, covering 5.47%), and quiescent regions (Qui, occupying 64.29%) (Fig. 2B). In general, promoters and TSS-proximal transcription regions exhibited the highest enrichment at TSS (Fig. S2A). Chromatin states were highly dynamic during follicular development, with 7%–25% of the genome changing state between adjacent stages (Fig. 2C and Fig. S2B).

We next examined whether histone modifications in GCs are reprogrammed during follicular development. We found that whole-genome chromatin accessibility and H3K4me3 levels remained low before the hierarchy stage but increased rapidly after follicle selection, maintaining high levels around ovulation (Fig. 2D and E). H3K27ac and H3K27me3 remained stable until sharply rising at F1 (the ovulation stage) (Fig. 2F and G). Meanwhile, H3K4me1 exhibited minimal overall change (Fig. 2H). These results reveal the specific dynamic patterns of different histone modifications during duck follicular development. Further, we focused on the effects of the histone modification and chromatin accessibility changes on gene expression during follicular development.

### H3K27ac changes are associated with the gene expression dynamics of GCs during follicular development

**Fig. 3:**
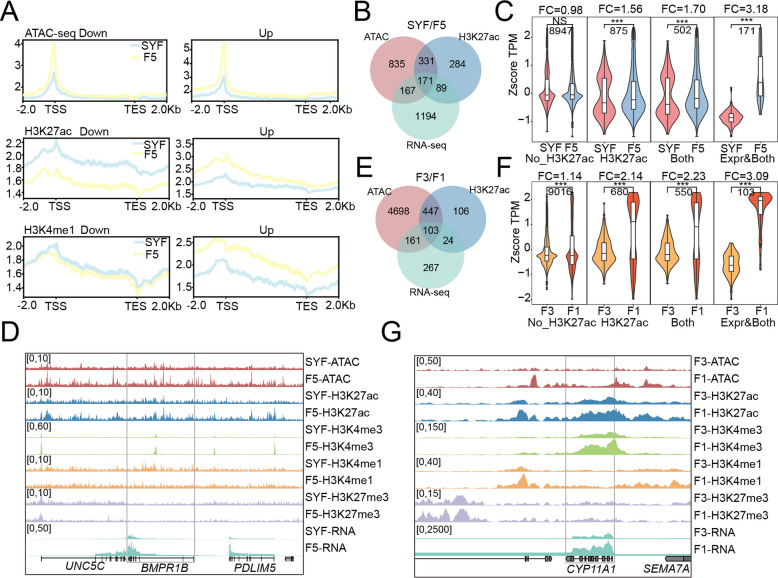
Influence of chromatin state alterations on gene expression dynamics in GCs. **A** Chromatin accessibility and histone modification profiles for down-regulated (left) and up-regulated (right) genes between SYF and F5 stages. **B** Overlap of chromatin accessibility (ATAC), H3K27ac modification, and gene expression (RNA-seq) between the SYF and F5 stages. ATAC and H3K27ac denote genes associated with significantly increased peaks, and RNA-seq represents differentially upregulated genes in the corresponding stage. **C** Expression levels of distinct gene sets derived from Fig. 3B. NO_H3K27ac indicates the genes without H3K27ac difference peaks in the two stages; H3K27ac represents the genes related to the H3K27ac differential upregulated peaks; Both indicates the genes related to the peaks where both chromatin accessibility and H3K27ac activity have increased; Expr & Both represents the upregulated DEGs where both chromatin accessibility and H3K27ac activity have increased. **D** and** G** Epigenetic profiles (ATAC-seq, H3K27ac, H3K4me3, H3K4me1, H3K27me3) and expression levels of two representative genes showing coordinated increases in chromatin accessibility, H3K27ac, and transcription between adjacent stages. **E** Overlap among increased chromatin accessibility, H3K27ac, and DEGs at the F3 and F1 stages.** F** Expression levels of various gene sets derived from Fig. 3E. The annotation is similar to Fig. 3C

Follicle selection and maturation are critical for avian egg production. In follicle selection, H3K27ac and H3K4me1 levels increased at upregulated genes and decreased at downregulated genes, whereas H3K27me3 showed the opposite trend (Fig. 3A and Fig. S3A). Chromatin accessibility and H3K4me3 were increased in both up-regulated and down-regulated genes (Fig. 3A and Fig. S3A). Notably, 3,313 differential H3K27ac peaks were detected between SYF and F5, while no significantly differential H3K4me1 peaks were observed (Fig. S3B). Among genes with increased H3K27ac (875 genes, FC = 1.56), 57.37% also exhibited increased chromatin accessibility (average FC = 1.70) (Fig. 3B). These co-increased regions were associated with higher expression levels (Fig. 3C and Fig. S3C). Genes with elevated chromatin accessibility, H3K27ac, and transcription levels (~ 19.54% of H3K27ac-upregulated genes, average FC is 3.18) included well-known follicle selection-related genes, such as *ESR1*, *BMPR1B*, and *WNT4* (Fig. 3D and Table S6) [10, 54]. These genes were enriched in pathways associated with hormone regulation, lipid metabolism, and enzyme-linked receptor protein signaling, all of which are crucial for follicle selection (Fig. S3D**)**.

Similar epigenetic dynamics were observed during follicular maturation (Fig. S3E). Here, 80.88% of genes with increased H3K27ac also showed elevated chromatin accessibility, and these co-modified genes exhibited higher expression (average FC = 2.23) (Fig. 3E, F and Fig. S3F). Genes upregulated in H3K27ac, chromatin accessibility, and transcription (~ 15.14% of H3K27ac-increased genes; average FC is 3.09) included *STAR* (an acute regulatory enzyme for steroid hormone synthesis) [55], *CYP11A1* (involved in cholesterol and steroid metabolism) [56, 57], and *TGFBR3* (a negative regulator of FSH secretion) (Fig. S3G and Table S6) [58, 59]. These genes were enriched in pathways related to collagen remodeling, ECM modification, and steroid hormone synthesis, all essential for follicle maturation (Fig. S3G). In conclusion, while chromatin accessibility facilitates a permissive environment for transcriptional competence, the synergistic enrichment of H3K27ac is associated with upregulation of stage-specific genes.

### Enhancers and SEs are correlated with gene expression during follicular development

**Fig. 4:**
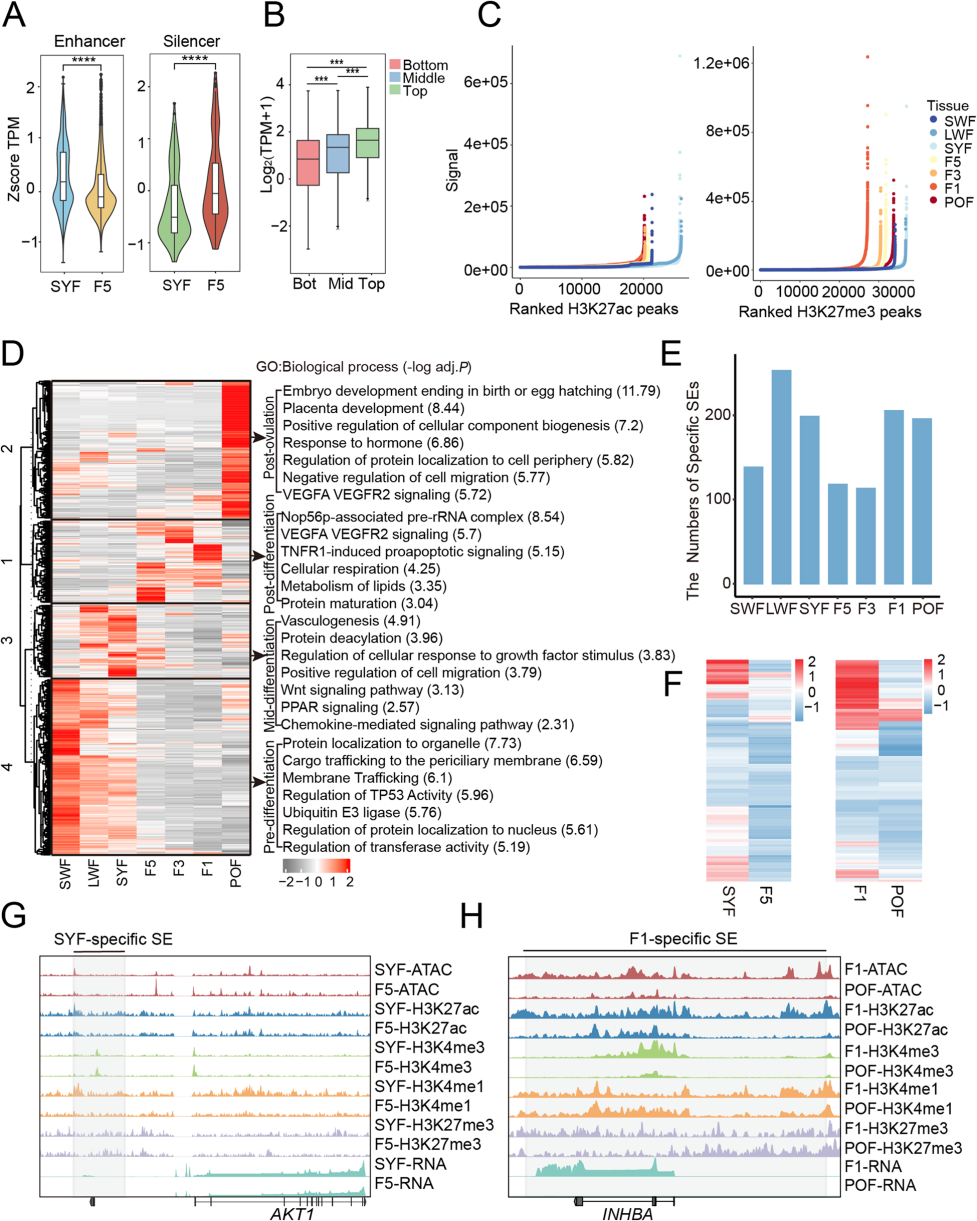
The potential functions of enhancers and SEs. **A** Expression levels of target genes linked to enhancers and silencers specific to the SYF stage (*P* < 0.001, Student's *t*-test). **B** Expression levels of genes associated with varying numbers of enhancers (Bottom = 1, Middle = 10–20, Top ≥ 50). **C** Identification of SEs (based on H3K27ac) and SSs (based on H3K27me3) using the ROSE algorithm at each stage. **D** Clustering of target genes associated with stage-specific SEs and their GO enrichment analysis. **E** Number of stage-specific SEs at each stage. **F** Heatmaps showing expression of genes linked to specific SEs between SYF and F5 (left), and F1 and POF (right). **G** and **H** Target gene of SYF-specific SE and F1-specific SE. Epigenetic signals from ATAC-seq, H3K27ac, H3K4me3, H3K4me1, and H3K27me3, along with gene expression levels

We identified the important role of H3K27ac in follicle selection and maturation. H3K27ac is a well-known marker for enhancers. The expression levels of target genes associated with stage-specific enhancers were significantly different among the six stages of follicular development, while the expression levels of target genes associated with silencers were significantly different in two stages, indicating that enhancers have a broader effect on gene expression levels (*P* < 0.001) (Fig. 4A and Fig. S4A). Genes associated with more enhancers exhibited higher expression levels (*P* < 0.01, Student’s *t*-test) (Fig. 4B). By integrating H3K27ac and H3K27me3 signals using the ROSE, we identified an average of 964 SEs and 1,040 SSs in GCs (Fig. 4C). Target gene expression showed a gradient of SE > TE > TS > SS (*P* < 0.01, Student’s *t* test) (Fig. S4B).

Follicular development entails GC proliferation and differentiation. To elucidate the functional role of SEs, we categorized non-redundant, stage-specific SEs. Their target genes (1,505 genes) were grouped into 4 clusters corresponding to pre-differentiation, mid-differentiation, post-differentiation, and post-ovulation stages (Fig. 4D and E). The average TSI (0.602) of stage-specific SEs was significantly greater than that of all genes (average TSI is 0.3730, Cliff's Delta estimate = 0.657), confirming their stage-specific activity.

The SEs in Cluster 3 were associated with the highly expressed genes in LWF, SYF, and F5 stages, and were enriched for Wnt signaling pathway, PPAR signaling pathway, and growth factor stimulation (Fig. 4D). Comparing SYF and F5, we found that SYF-specific SEs were associated with 336 SYF-specific genes, including *AKT1*, *INHBB*, *POMC*, and *ACAT2* (Fig. 4F and G). These genes were enriched in cell cycle, differentiation, transcription, and signal transduction, such as cell differentiation, cytokine stimulus, regulation of the Notch signaling pathway, and VEGFA-VEGFR2 signaling pathway (Fig. S4C). These pathways may maintain the follicle in a viable but undifferentiated state in preparation for selection [60]. Together, these findings suggest that SEs are closely linked to follicle selection.

To assess SE roles in ovulation, we identified F1-specific SEs linked to 209 genes, including *INHBA*, *IGF1R*, *BMPR1B*, and *ELOVL5* (Fig. 4H). These genes were involved in biological processes like hormone regulation, steroid metabolism, energy metabolism, and protein folding, which were crucial for ovulation (Fig. S4D). In summary, our data emphasize that enhancers and SEs show a strong association with the expression of genes related to follicular development.

### 3D structural changes play a modulatory role in transcriptional regulation during follicular development

**Fig. 5:**
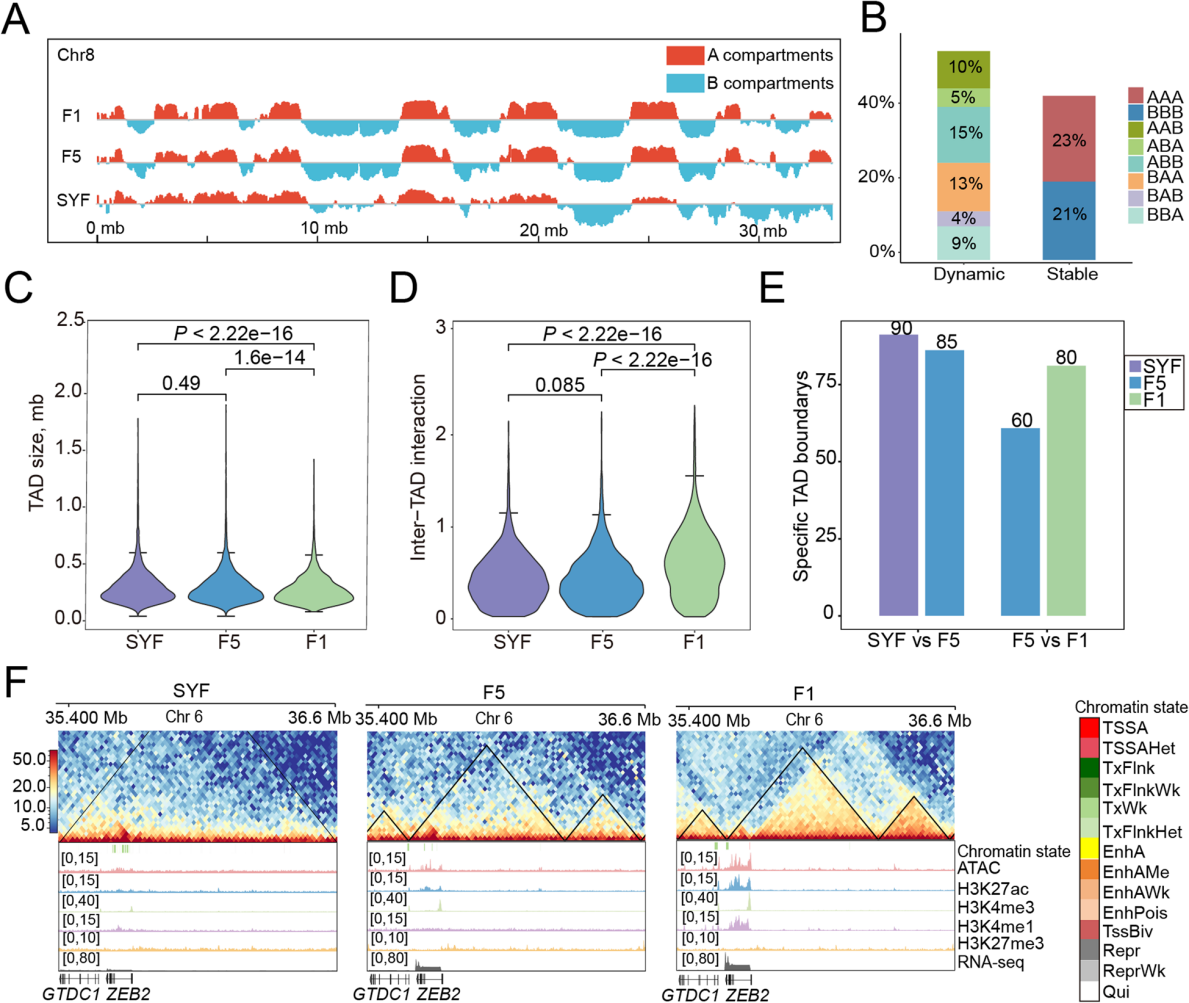
Three-dimensional structural dynamics of ovarian GCs during folliculogenesis. **A** Chromosomal compartment transitions on chromosome 8. **B** Genomic length and proportion of stable and dynamic compartments. **C** Box plots illustrating the size of TADs (Mb) in GCs during folliculogenesis. The numbers in the figure represent the *P*-values obtained through the *t*-test. **D** Intra-TAD contacts of the 3 developmental stages. **E** The number of stage-specific TAD boundaries. **F** A representative gene at a differential TAD boundary during follicular development. Upper panel: Hi-C contact heatmap for the genomic region containing *ZEB2*. Lower panel: Epigenetic signals from ATAC-seq, H3K27ac, H3K4me3, H3K4me1, H3K27me3, along with gene expression levels

The 3D structure of chromatin can provide the regulatory function of enhancers. We performed high-resolution Hi-C sequencing at 3 follicular stages (SYF, F5, and F1) and constructed genome-wide chromatin interaction maps (Fig. S5A). Analysis of A/B compartment distributions revealed that 44% of genomic compartments remained stable across stages, with 49%–52% consistently residing in the active A compartment (Fig. 5A). Extensive compartment switching was observed across the 3 stages, with unidirectional transitions from A to B at 25%, B to A at 22%, and transient ABA and BAB switches at 9% (Fig. 5B). Although compartmentalization is dynamic, significant differential gene expression linked to these transitions was detected only at the F5 and F1 stages (*P* < 0.01, Student’s *t* test) (Fig. S5B). This suggests that compartmentalized rearrangement contributes to a limited extent to the global transcriptional changes observed during follicular development. Functional enrichment analysis demonstrated that genes within regions switching from B to A compartments were primarily involved in cell proliferation and differentiation, tissue morphogenesis, and cell adhesion (Fig. S5C). Besides, key follicle maturation genes, including *LHCGR* (encoding luteinizing hormone) [61], *BMPR2* [62], and *EGFA* [63, 64], were specifically located in the A compartment at the F5 and F1 stage (Fig. S5D).

At a resolution of 20 kb, an average of 3,773 TADs were detected in duck GCs, with an average size of ~ 296 kb (Fig. 5C). Notably, 97.9% of TADs were present at all stages, indicating the conservation of TAD structures during follicular development (Fig. 5E). While intra-TAD interaction strengths showed no significant change during follicle selection, they increased significantly from the F5 to the F1 stage (*P* < 0.01 Student’s *t*-test) (Fig. 5D). Compared to F5, the SYF stage exhibits 90 specific TAD boundaries, within which 98 genes were significantly enriched (Fig. 5E and Table S7), including *BMP15*, *CDH8*, and *CSMD3*. In addition, the comparison between the F1 and F5 reveals 60 specific TADs, with 54 genes significantly enriched in these specific boundaries in F5 (Fig. 5E and Table S7), including *ERBB4*, *EP300*, and *STAG2*. Genes linked to these dynamic boundaries were enriched in follicular development pathways, including regulation of the MAPK cascade, PI3K events in *ERBB4* signaling, and the estrogen signaling pathway (Table S8). While genes near stage-specific TAD boundaries showed no global shifts in expression level compared to the genomic background (Fig. S5E), we observed that specific boundaries flanking key follicular genes underwent precise local reorganization. For example, *ZEB2* (which regulates intercellular adhesion and cytoskeletal rearrangement via E-cadherin) [65] acquired a TAD boundary, and its expression increased in both the F1 and F5 stages (Fig. 5F). This suggests that in duck folliculogenesis, the 3D genome functions as a relatively stable structural scaffold that accommodates highly targeted, stage-specific regulatory fine-tuning, rather than acting as a global driver of transcription.

### A multi-layered *cis*-regulatory landscape revealed by integrated multi-omics

**Fig. 6:**
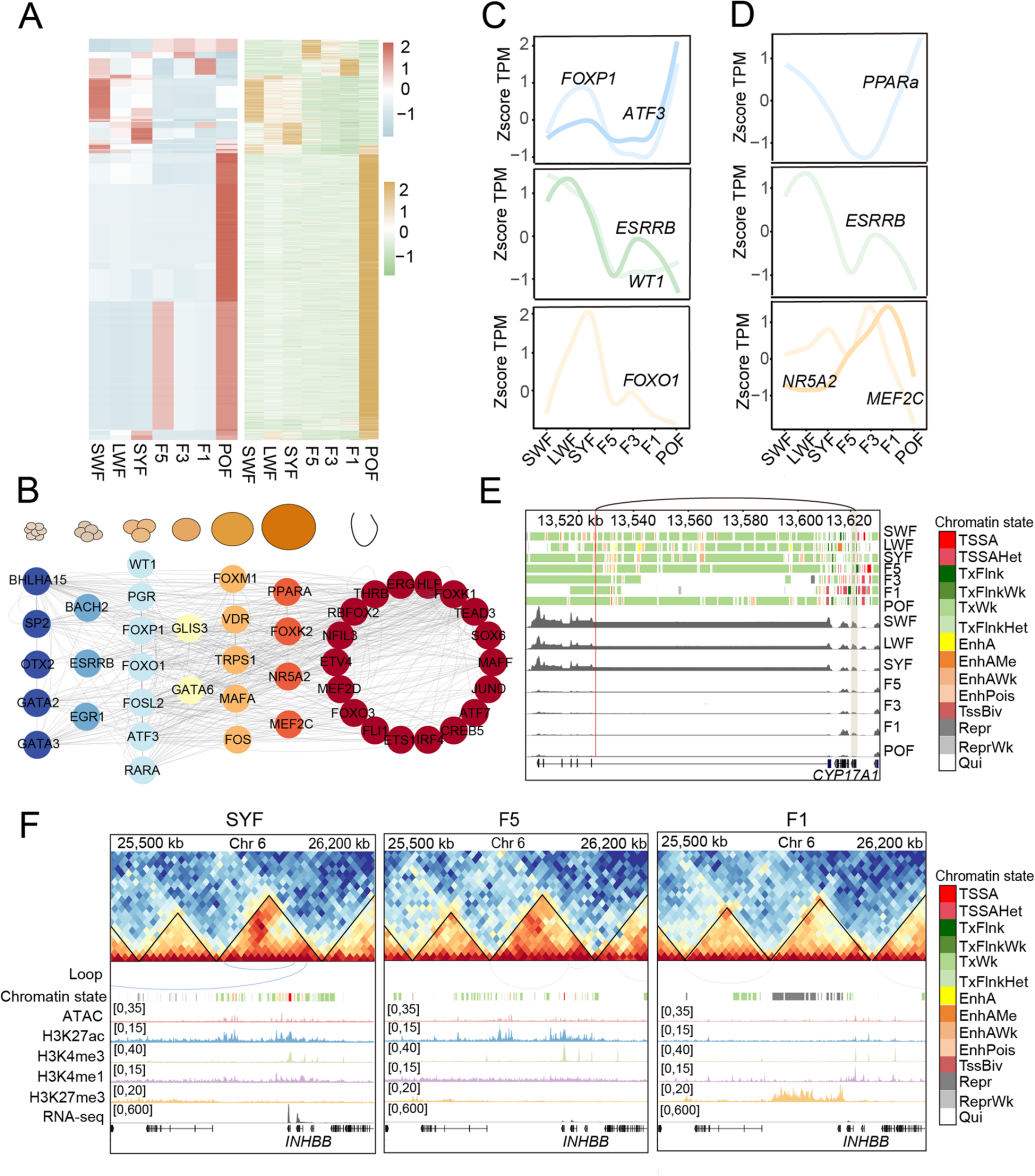
3D *cis*-regulatory network during follicular development. **A** Heatmap depicting expression patterns between TFs and their target genes. Each row in the heatmap represents either a TF or a gene. **B** Regulatory network of core TFs involved in follicular development, arranged from left to right by developmental stage (When a TF is expressed at multiple stages, the stage with the highest expression level is depicted). **C** Expression patterns of representative key TFs at the SYF stage. **D** Expression patterns of representative TFs at the F1 stage. **E** The enhancer of the variations related to egg production regulates *CYP17A1.* Top: chromatin states across 7 stages. Bottom: *CYP17A1* expression levels across stages. The red line represents the variation site, and the lightyellow part represents the gene. **F** Chromatin interaction heatmaps, enhancer-gene pairs, chromatin state, trace maps of ATAC-seq, CUT&Tag, and RNA-seq signals at the indicated genomic loci. The blue line represents loops

To characterize the transcriptional control of duck folliculogenesis, we constructed putative GRNs by integrating TF motif enrichment and enhancer-gene associations. The inferred GRN comprised 8,132 enhancers, 232 TFs, and 3,376 genes (Table S9). As expected, proposed TFs and putative target genes showed similar transcriptional profiles during follicular development (Fig. 6A). Through retaining the differential TF-mediated regulatory relationships in different DEG clusters, 46 core proposed TFs related to the follicle development were identified (Fig. 6B).

To elucidate TF coordination, we examined the interaction network among proposed core TFs (Fig. 6B and Table S10). The proposed TFs implicated in follicle selection (such as *FOXP1*, *FOXO1*, *ESRRB*, *ATF3*, and *WT1*) were significantly more highly expressed at the SYF stage than at F5 (*P* < 0.01 Student’s *t*-test) (Fig. 6C). These TFs regulate follicle selection by participating in the proliferation, differentiation, and apoptosis of GCs. TFs related to ovulation included *PPARα*, *MEF2C*, *NR5A2* [66], and *ESRRB*. They showed the highest expression at the F1 stage, suggesting a direct association with impending ovulation (*P* < 0.01 Student’s *t*-test) (Fig. 6D).

Previous studies identified 248 loci related to egg-laying traits through four consecutive generations of GWAS data of Pekin ducks [11]. 27 loci overlap with GCs enhancers and potentially target 16 genes (Table S11). For instance, rs7:13532237 (*P* = 4.26 × 10⁻^6^) coincides with an enhancer (7:13532200–13532800) that may influence follicular development by regulating *CYP17A1*, a key enzyme in sex hormone synthesis (Fig. 6E) [67].

By integrating epigenomics, 3D genomics, and transcriptomics data, this study reveals 3 *cis*-regulatory mechanisms: (1) Proximal enhancer activity: 8,132 proximal regulatory elements, located an average of 82,248 bp from their promoters, regulate 3,376 target genes (Table S9); (2) Distal enhancer activity via chromatin loop: 9,788 distal regulatory elements (average distance of 359,411 bp) are linked to 4,326 target genes through chromatin interactions (Table S12). A representative example is *INHBB*, a gene known to regulate GC proliferation and steroidogenesis [68]. At the SYF and F5 stages, specific enhancers were identified to potentially interact with the promoter region of *INHBB* through chromatin loops, and are consistent with their elevated transcriptional levels (Fig. 6F). (3) TAD structure reorganization: Alterations in TAD boundaries correlate with the expression of 149 genes (Table S7).

### Comparison of follicle development in birds and mammals

**Fig. 7:**
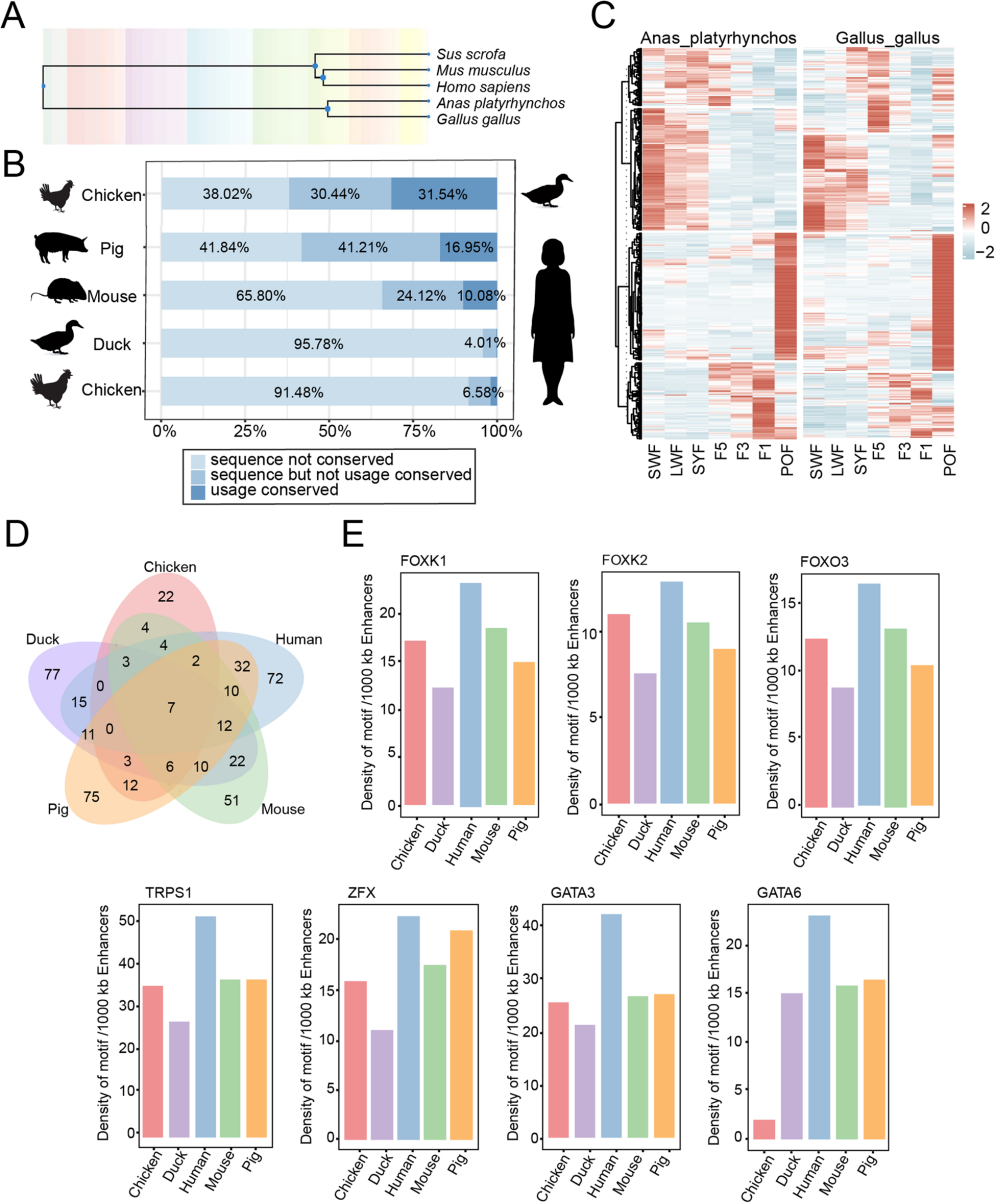
Comparison of conserved follicular development between avian and mammalian species. **A** Evolutionary relationships among human, pig, mouse, chicken, and duck.** B** Sequence conservation of enhancers (liftOver to each genome) and functional conservation across five species. **C** Heatmap comparing expression patterns of enhancer target genes in chicken and duck. **D** Venn diagram illustrating the conservation of TFs across the five species. **E** Density of conserved TFs per 1,000 kb of enhancer regions. The 7 TFs universally conserved across all five species are highlighted

The process of follicular development in birds and mammals displays both similarities and differences. We performed a cross-species epigenomic comparison to assess the conservation of enhancers and TFs across duck, chicken, human, mouse, and pig (Fig. 7A). Within birds, 30.44% of duck enhancer sequences were conserved in chicken. Among these, 31.54% also showed functional conservation (Fig. 7B). Furthermore, genes targeted by enhancers with conserved functions exhibited similar expression patterns (Fig. 7C). These genes were enriched in pathways, such as hormone response, calcium-mediated signal transduction, and neural projection development (Fig. S6A). Our analysis suggests that enhancer conservation is higher within the mammalian lineage compared to the bird-mammal (Fig. 7B). This pattern likely reflects both the rapid evolutionary turnover of regulatory sequences and the technical challenges of aligning non-coding regions across vast evolutionary distances.

We next examined the conservation of TF binding. We identified 7 conserved TFs across 5 species, 13 conserved TFs in birds, 24 conserved TFs in mammals, and 10 mammal-specific TFs (Fig. 7D and Fig. S6B). Both bird and mammalian conserved TFs were enriched in pathways such as gonad development, hormone response, and glucose homeostasis maintenance (Fig. S6C and D), which preliminarily indicates the conservation of these pathways in follicular development. Finally, we compared the genome-wide distribution of conserved motifs (*GATA3*, *GATA6*, *FOXK1*, *FOXK2*, *FOXO3*, *TRPS1*, and *ZFX*) across birds and mammals. The relative density of these motifs was different across species (Fig. 7E). This suggests that while conserved TF binding motifs are present across species, their distribution is not uniform. These results may provide a preliminary explanation for the diversity of ovulation regulation among different species.

## Discussion

In this study, we systematically analyzed RNA-seq, ATAC-seq, CUT&Tag, and Hi-C data across duck follicular development, constructing the comprehensive three-dimensional *cis*-regulatory landscape of avian follicle development. Although epigenetic research in birds has expanded, studies on the epigenetic regulation of follicular development remain scarce and lack systematic depth. The gene expression during follicular development is continuous and jointly regulated by chromatin accessibility and histone modifications [20]. ATAC-seq and CUT&Tag analyses were performed with biological replicates (*n* = 2) following ENCODE guidelines. Although replicates demonstrated high internal consistency (Pearson correlation > 0.82), we acknowledge that this sample size limits the statistical power to detect subtle quantitative differences. Consequently, genomic regions reported as unchanged should be interpreted as showing no statistically significant difference within the sensitivity limits of this study, rather than being definitively static. To mitigate this, we have prioritized the interpretation of robust, high-confidence differential peaks throughout the analysis.

Our results demonstrate that the remodeling dynamics of different histones vary across distinct stages of follicular development. For instance, H3K27ac remains stable until a sharp increase at the F1 stage. Notably, dynamic H3K27ac remodeling and its sustained enrichment in preovulatory follicles have also been reported in mice [52], suggesting an evolutionary conservation of this epigenetic strategy to drive final maturation. However, unlike the cyclic cohort recruitment in mammals, the unique hierarchical organization of avian follicles likely requires distinct, stage-specific epigenetic programs to maintain the precise sequential ovulation characteristic of birds. This work provides the first comprehensive profile of histone modifications throughout duck follicular development.

Follicle selection and maturation are pivotal events. During these two processes, H3K27ac and H3K4me1 exhibit varying degrees of elevation in upregulated genes, and downregulated genes show a decrease. The chromatin accessibility and H3K4me3 were increased in both up-regulated and down-regulated genes. Our results highlight the distinct yet overlapping roles of different epigenetic marks. While H3K4me3 and ATAC-seq peaks appear to serve as stable indicators of promoter identity, they show significant redundancy and remain largely unchanged across follicular stages. In contrast, the synergy between H3K27ac and chromatin accessibility is much more dynamic. We found that open chromatin alone is insufficient for high-level gene expression; rather, it is the subsequent recruitment of H3K27ac that acts as the primary role of stage-specific transcriptional surges. This hierarchical relationship explains how the duck GCs regulatory landscape is primed and then precisely activated.

There are two possible mechanisms of enhancer regulation of genes: First, *cis*-regulatory mechanisms, such as SNP or other DNA sequence variation may directly change the sequence near the enhancer, or indirectly affect enhancer activity by changing local chromatin structure, thus affecting the binding ability of TFs; The second is the trans-regulatory mechanism, such as the expression level of TFs at different developmental stages may affect their binding and regulation of enhancers, resulting in differences in enhancer activity. To explore potential regulatory mechanisms, we constructed predicted GRNs by integrating epigenomic and transcriptional data. It not only provides insights into the known mechanisms of genes promoting follicular development, such as *FOXO1* [69, 70], *NR5A2* [66], and *WT1*, but also aids in identifying new key TFs that could enhance follicular development. We identified 46 core TFs essential for follicular development, whose stage-specific expression patterns suggest distinct temporal roles.

We acknowledge that our cross-species analysis of enhancer and TF conservation is subject to methodological limitations, particularly the difficulty in detecting functionally conserved enhancers that lack sequence homology (enhancer turnover). Within this context, we identified 7 TFs (*GATA3* [71, 72], *GATA6* [73, 74], *FOXK1 *[75], *FOXK2*, *FOXO3* [76, 77], *TRPS1*, and *ZFX* [78]) whose binding motifs are conserved between mammals and birds. Their non-uniform genomic distribution may reflect species-specific adaptations in regulatory architecture.

While our integrated analysis offers a detailed regulatory map, it is important to consider these findings within the context of certain limitations. First, the sample collection protocol did not record the precise position of the egg within the oviduct, which limits the resolution of our data regarding the ultrafine regulatory shifts in the final hours preceding ovulation. Future studies integrating in vivo temporal tracking will be essential to further refine the chronobiology of duck follicle maturation. Second, the links identified between chromatin accessibility, histone modifications, and gene expression are primarily built on statistical associations and computational predictions. Although we applied rigorous filtering to ensure high-confidence results, these data points reflect correlations rather than direct causality. Furthermore, as this study focused on establishing the multi-omics landscape, we did not perform functional validations such as CRISPR-Cas9 editing or enhancer reporter assays. Moving forward, experimental verification of these predicted networks will be essential to fully confirm the biological roles of the candidate genes and enhancers identified here.

## Conclusion

In conclusion, we first provide a three-dimensional *cis*-regulatory map of duck follicle development, identifying key enhancers, TFs, and the interaction regulatory network during bird folliculogenesis. We further reveal TFs conserved between birds and mammals during follicular development. These findings offer a valuable resource for understanding the mechanisms through which GCs support follicular development.

## Supplementary Information

Additional file 1: Fig. S1. Data summary. Fig. S2. Prediction and characterization of chromatin states during follicular development. Fig. S3. Impact of chromatin state alterations on the gene expression dynamics of GCs. Fig. S4. Enhancers and SEs exert functional roles in follicular development. Fig. S5. 3D structure and regulation of GCs during follicle formation. Fig. S6. Conserved TFs and their functions in avians and mammals.

Additional file 2: Table S1. The sequence information for each sample of RNA-seq. Table S2. The summary of data quality of all the CUT&Tag of epi-marks and ATAC-seq data sets of the duck. Table S3. The number and proportion of DEGs between the two stages. Table S4. The significant differences in the number of DEGs between developmental stages. Table S5. The number, genome coverage, and size of chromatin state in the follicle. Table S6. Overlap between chromatin accessibility, H3K27ac, and DEGs. Table S7. Differential TADs target genes during follicular development. Table S8. The enriched pathways of genes associated with specific boundaries. Table S9. Specific Enhancer-TF-Gene pairs at adjacent stages. Table S10. The core TF interaction in GRN and the TF interaction predicted by STRING. Table S11. The variations related to egg production are located in enhancers and target genes. Table S12. Specific long-range enhancer-gene interaction.

## Data Availability

The RNA-seq, Hi-C, ATAC-seq, and CUT&Tag data generated in this study have been deposited in the SRA database under accession code PRJNA1254901. The codes and scripts used for data processing and analyses are available on GitHub through the following link: https://github.com/lily585/duck_GCs_Paper_codes.git.

## Abbreviations

ATAC-seq: Assay for transposase-accessible chromatin using sequencing
CUT&Tag: Cleavages under targets and tagmentation sequencing
DEGs: Differentially expressed genes
GCs: Granulosa cells
GRN: Gene regulatory network
Hi-C: High-throughput chromosome conformation capture
LWF: Large white follicle
POF: Post-ovulatory follicle
SEs: Super-enhancers
SSs: Super-silencers
SWF: Small white follicle
SYF: Small yellow follicle
TADs: Topologically associating domains
TEs: Typical enhancers
TFs: Transcription factors
TSs: Typical silencers
