# Supplementary material for: Comprehensive multi-omics reveals dynamic chromatin changes and gene regulatory networks during duck folliculogenesis
Source: J Anim Sci Biotechnol. 2026 Apr 30;17:79. doi: 10.1186/s40104-026-01393-z (PMC13134274; doi:10.1186/s40104-026-01393-z)
Supplement: Supplementary file 1 — Additional file 1: Fig. S1. Data summary. Fig. S2. Prediction and characterization of chromatin states during follicular development. Fig. S3. Impact of chromatin state alterations on the gene expression dynamics of GCs. Fig. S4. Enhancers and SEs exert functional roles in follicular development. Fig. S5. 3D structure and regulation of GCs during follicle formation. Fig. S6. Conserved TFs and their functions in avians and mammals. [file 40104_2026_1393_MOESM1_ESM.pdf]

Supporting Information

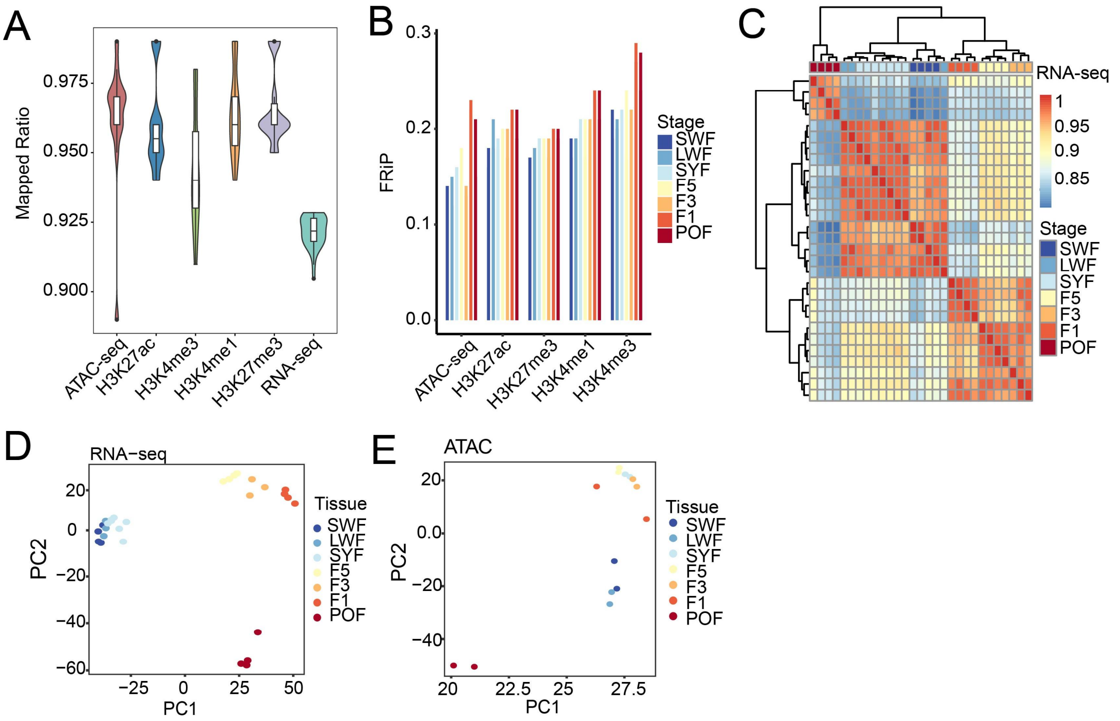

Fig. S1 Data summary. **A** The ratio of reads mapped to the mallard duck genome to the total reads in each library. **B** The average fraction of reads in peaks (FRiP) for epigenetic marks across 7 stages. **C** Correlation heatmap of the transcriptomic samples. **D** PCA analysis of RNA-seq data. **E** PCA analysis of ATAC-seq data.

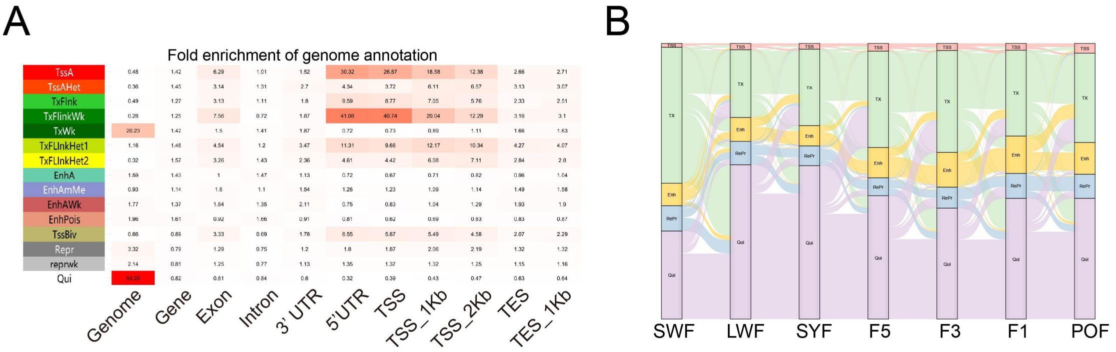

Fig. S2 Prediction and characterization of chromatin states during follicular development. **A** An average enrichment size of chromatin states annotated in the genome. **B** The flow of chromatin states between adjacent stages in the morula diagram.

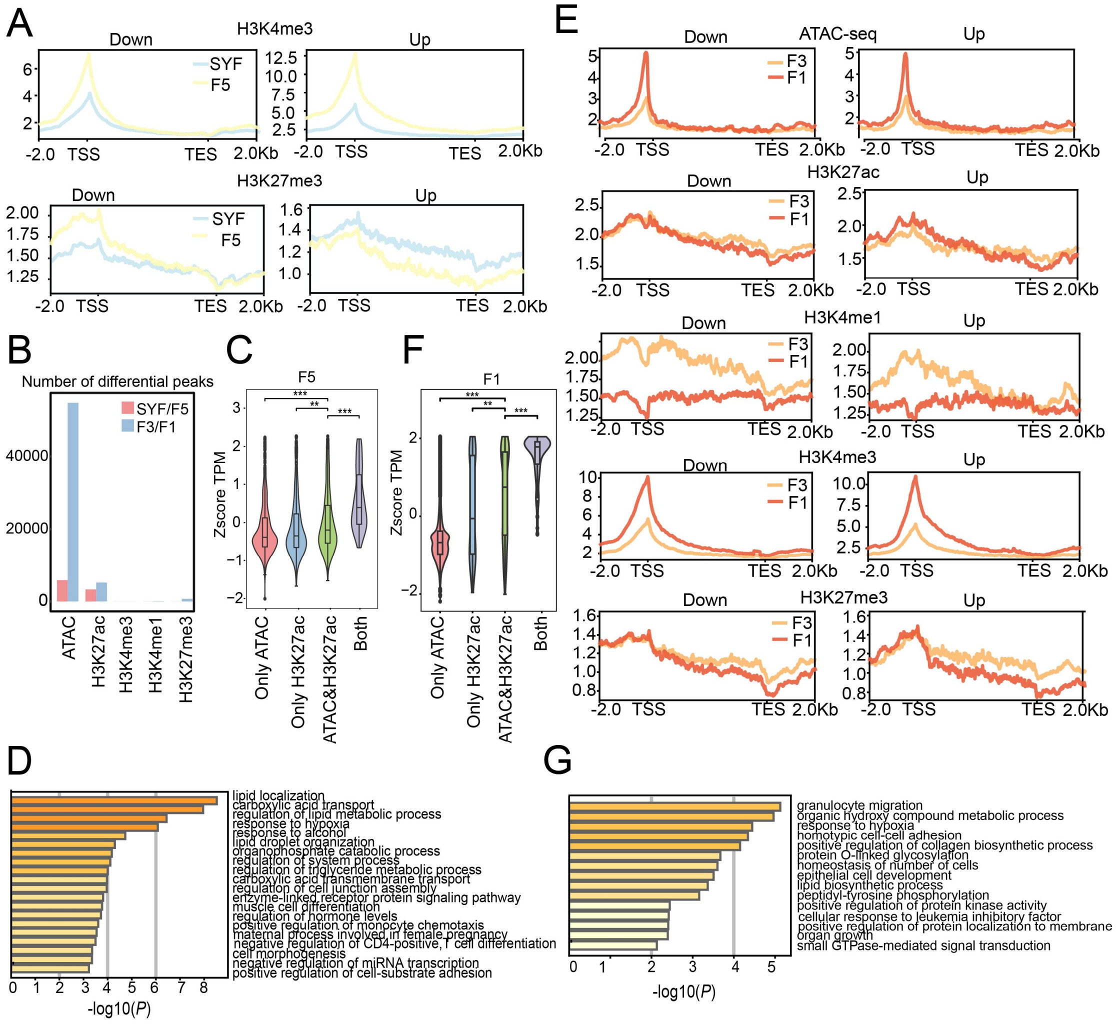

Fig. S3 Impact of chromatin state alterations on the gene expression dynamics of GCs. **A** H3K4me3 and H3K27me3 signals for down-regulated and up-regulated genes between SYF and F5 stages. **B** Number of differential peaks between SYF/F5 and F3/F1 stages. **C** Expression levels of distinct gene sets at F5. "Only ATAC" denotes genes associated with significantly increased chromatin accessibility, and "Only H3K27ac" denotes genes associated with increased H3K27ac at the F5 stage. "ATAC&H3K27ac" indicates genes related to the peaks where both chromatin accessibility and H3K27ac activity have increased. "Both" represents the upregulated DEGs where both chromatin accessibility and H3K27ac activity have increased. **D** GO and KEGG enrichment results for genes with elevated chromatin accessibility,

H3K27ac, and transcription levels between SYF and F5 stages. **E** Chromatin accessibility and histone modification profiles for down-regulated and up-regulated genes between F3 and F1 stages. **F** Expression levels of distinct gene sets at F1. The meaning of the gene set is similar to that of Fig. S3C. **G** GO, and KEGG enrichment results for genes with increased chromatin accessibility, H3K27ac, and transcription levels between F1 and F3 stages.

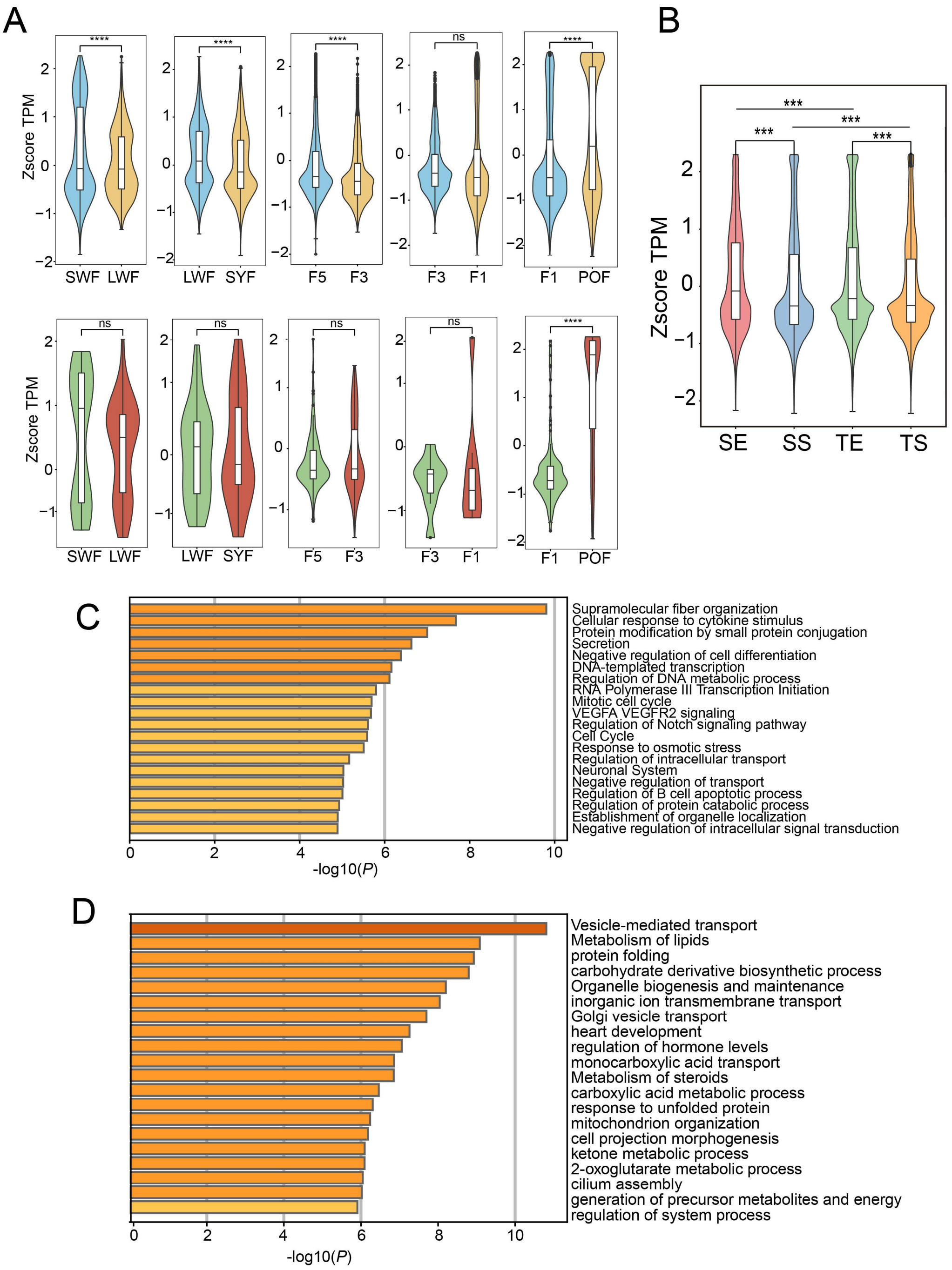

Fig. S4 Enhancers and Super-enhancers exert functional roles in follicular development. **A** Expression levels of enhancers and silencers target genes that are specifically present in adjacent developmental stages. **B** Violin plots showing the expression levels of SEs, TEs, SSs, and TSs target genes. **C-D** GO and KEGG enrichment analysis of SEs targeting genes specifically present at the SYF (C) and F1 stage (D).

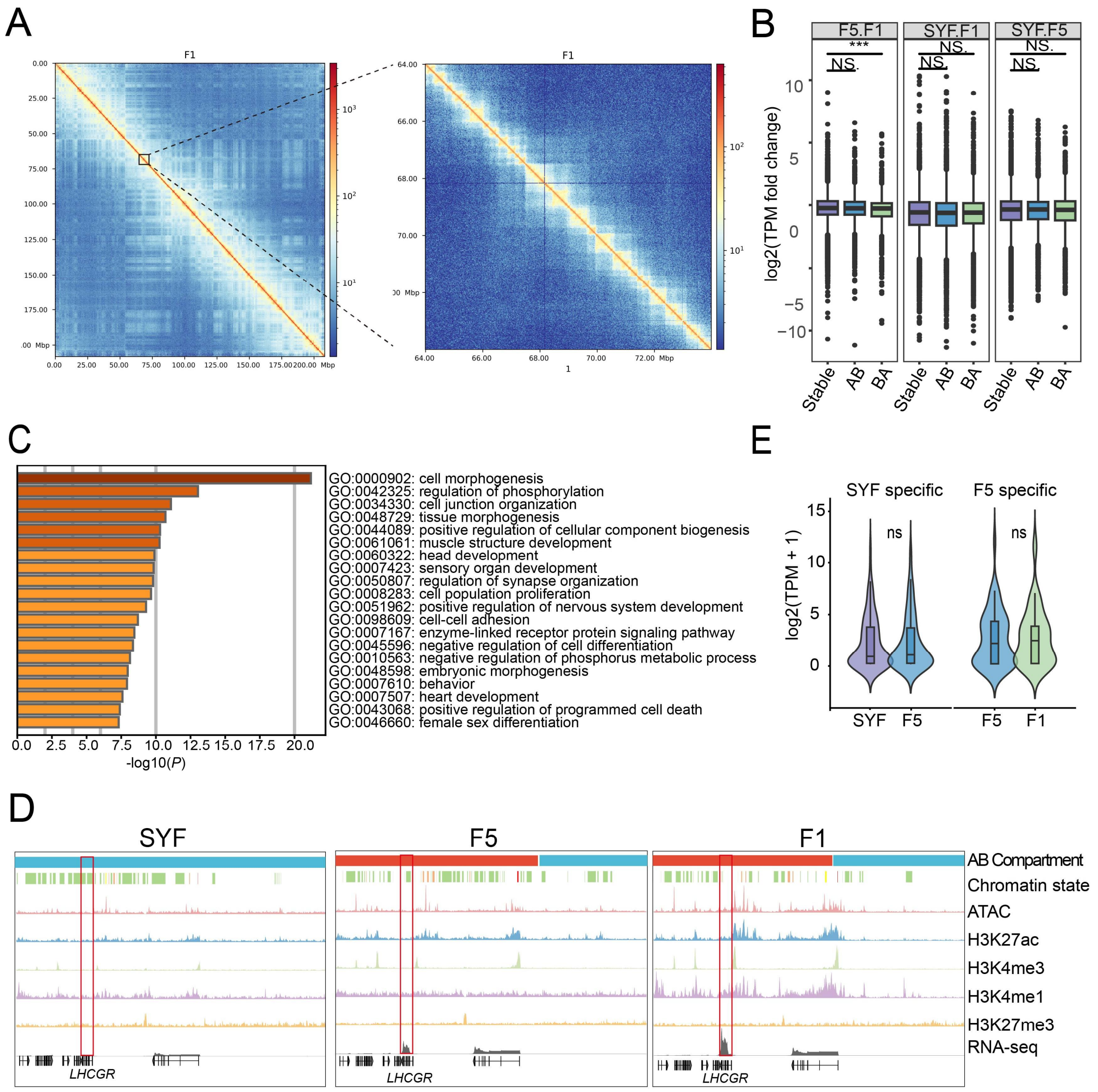

Fig. S5 3D structure and regulation of GCs during follicle formation. **A** Contact heatmap of chromosome 1 at 150 kb and 20 kb resolution. **B** Differential gene expression between stable and compartment-switching compartments. **C** GO and KEGG enrichment analysis of genes in the BAA transient switching compartments. **D** Compartment, histone signals, and expression levels of *LHCGR* during follicular development. The red represents A compartments, and the blue represents B Compartments. **E** Comparison of gene expression levels at specific TAD boundaries.

A

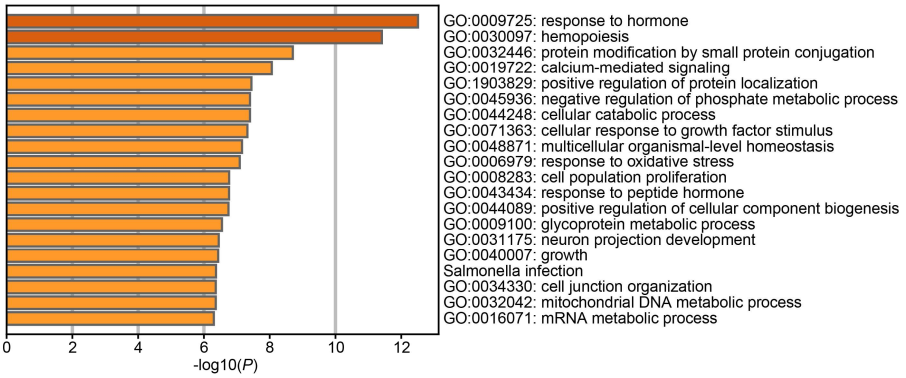

B

Bird Conserved	Mammals Conserved	Mammal-Specific	Species-Conserved
FOXO3	*FOXK1*	*ZIC2*	*GATA3*
FOXK1	*FOXO1*	*HIC1*	*GATA6*
RBFOX2	*ZIC2*	*MAX*	*FOXK1*
FOXF1	*HIC1*	*NF1*	*FOXK2*
TRPS1	*FOXK2*	*RUNX*	*FOXO3*
FOXP1	*MAX*	*FOS*	*TRPS1*
ZNF711	*NF1*	*JUNB*	*ZFX*
GATA6	*MAFA*	*FOSL2*	
FOXK2	*GATA6*	*BATF*	
GATA3	*GATA1*	*ATF3*	
SOX6	*GATA2*		
RXR	*NR5A2*		
ZFX	*GATA4*		
	FOS		
	GATA3		
	SF1		
	TRPS1		
	JUNB		
	FOSL2		
	ESRRB		
	BATF		
	ATF3		
	FOXO3		
	ZFX		

C

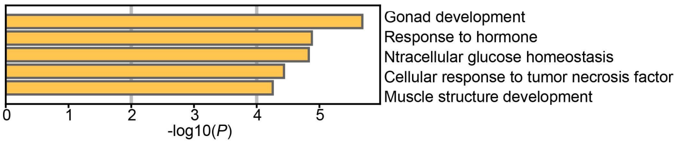

D

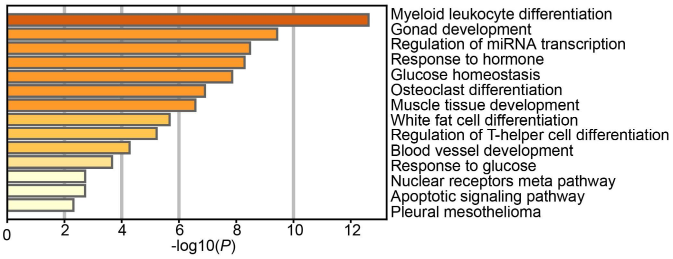

E

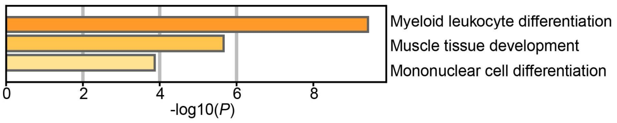

Fig. S6 Conserved TFs and their functions in avians and mammals. **A** GO and KEGG enrichment analysis of homologous target genes of functionally conserved enhancers in birds. **B** Conserved and species-specific TFs in birds and mammals. **C** GO and KEGG enrichment analysis of conserved TFs in birds. **D** GO and KEGG enrichment analysis of conserved TFs in mammals. **E** GO and KEGG enrichment analysis of mammal-specific TF.
